# Why People Enter and Embrace Violent Groups

**DOI:** 10.3389/fpsyg.2020.614657

**Published:** 2021-01-07

**Authors:** Ángel Gómez, Mercedes Martínez, Francois Alexi Martel, Lucía López-Rodríguez, Alexandra Vázquez, Juana Chinchilla, Borja Paredes, Mal Hettiarachchi, Nafees Hamid, William B. Swann

**Affiliations:** ^1^Department of Social and Organizational Psychology, Faculty of Psychology, Universidad Nacional de Educación a Distancia, Madrid, Spain; ^2^ARTIS International, St. Michaels, MD, United States; ^3^Department of Psychology, University of Texas at Austin, Austin, TX, United States; ^4^Department of Psychology, Faculty of Psychology, Universidad de Almería, Almería, Spain; ^5^Department of Theory and Analisys of Comunication, Faculty of Sciences of Information, Universidad Complutense de Madrid, Madrid, Spain; ^6^InReach Global, Centre for Psycho-Social Research & Training, Colombo, Sri Lanka

**Keywords:** radicalization, terrorism, identity fusion, collective identity, social influence

## Abstract

We distinguish two pathways people may follow when they join violent groups: compliance and internalization. Compliance occurs when individuals are coerced to join by powerful influence agents. Internalization occurs when individuals join due to a perceived convergence between the self and the group. We searched for evidence of each of these pathways in field investigations of former members of two renowned terrorist organizations: the Liberation Tigers of Tamil Eelam (LTTE) (Study 1) and Islamist radical groups (Study 2). Results indicated that ex-fighters joined LTTE for reasons associated with both compliance and internalization but that ex-fighters joined Islamist radical groups primarily for reasons associated with internalization. When compliance occurred, it often took the form of coercion within LTTE but involved charismatic persuasion agents within Islamist groups. This evidence of systematic differences in the reasons why fighters enter violent groups suggests that strategies for preventing radicalization and fostering de-radicalization should be tailored to particular groups.

## Introduction

Violent extremism and terrorism pose a growing threat to peace and security worldwide. To reduce this threat, the UN has recently declared 2020–2030 the Decade of Action. A top priority is fighting violent extremism through the adoption of systematic preventive measures (United Nations, 2006). Identifying these measures requires understanding the fundamental issue of why people join violent groups. Although previous researchers have developed several distinct classification systems for organizing the reasons people join violent groups (e.g., Bjørgo, 2011; Cottee and Hayward, 2011; Hafez and Mullins, 2015), no single formulation has won widespread acceptance among researchers.

The present research aims to contribute to understanding why people join violent groups in three ways. First, we draw on the attitude change literature (e.g., Kelman, 1952, 1958; Bagozzi and Lee, 2002) to distinguish two general pathways through which people may come to join violent groups: compliance and internalization. Second, we elaborate three situationally-driven sub-pathways that give rise to compliance (charismatic persuasion agent, propaganda, and coercion) and three identity-driven sub-pathways that give rise to internalization (personal, relational, and collective identities). Third, we assess the applicability of our formulation in understanding why members of two violent terrorist organizations joined the group. Specifically, in Study 1 we used semi-structured interviews to directly assess the experience of ex-members of the Liberation Tigers of Tamil Eelam (LTTE), a militant terrorist organization of Sri Lanka. In Study 2 we analyzed the life stories of former Islamist radicals who were ex-members of violent jihadist groups. Prior to introducing our formulation, we review past attempts to understand the roots of terrorism.

## Why People Join Violent Terrorist Groups: Basic Personal Needs, Shared Realities, and the Desire for Immersion Through Identity Fusion

Previous studies have devoted considerable attention to the question of why people join violent groups (e.g., Horgan, 2005; Moghaddam, 2005; Wiktorowicz, 2005; Newman, 2006; McCauley and Moskalenko, 2008; Sánchez-Cuenca and de la Calle, 2009; Borum, 2011; Campana and Lapointe, 2012; Horowitz, 2015; Scull et al., 2020). Intuitively, one might believe that alignment with terrorist groups is explained by radical ideology.

This commonsense assumption collides with the fact that most people holding radical ideas do not actually engage in terrorism, and many terrorists are not completely radicalized (Bjørgo, 2011). Radicalization does not inevitably lead to violence and terrorism, even though it can facilitate them (Bjørgo and Horgan, 2009). After all, previous research indicates that attending religious services (thought to enhance coalitional commitment) is a more powerful predictor of support for suicide attacks than religious devotion (Ginges et al., 2009). Therefore, radical worldviews are only one among many potential causes of joining violent terrorist groups (Kruglanski and Fishman, 2009).

With such distinctions in mind, Borum (2011) defines *radicalization* as “the process of developing extremist ideologies and beliefs.” This development of ideology is conceptually different from actual extremist acts, which Borum defines as *action pathways*, or “the process of engaging in terrorism or violent extremist actions” (p. 9). Our current focus is not the adoption of extremist ideologies *per se*, but the reasons that motivated former terrorists to join and support a terrorist group in the first place.

In line with the foregoing reasoning, the 3N model (Kruglanski et al., 2018; Bélanger et al., 2019; Lobato et al., 2020) identifies three general drivers of joining violent groups: need, narrative and network. According to this perspective, group membership can satisfy basic needs such as the need to feel valued and to be respected by others (Kruglanski et al., 2018). Different factors such as personal failures, interpersonal rejection, individual or collective grievances, or social alienation can induce a loss of personal significance through the loss of a compelling life narrative and the corresponding sense of purpose. To restore it, people may join groups that offer them a sense of purpose paired with feelings of camaraderie (Bélanger et al., 2019). Therefore, through joining such groups, individuals can address the basic need to be respected by others, they can establish a new narrative that gives their life meaning, and they also can experience the social benefits of being part of a network of people.

Groups do not operate in an ideological vacuum, but promote a shared reality (Hardin and Higgins, 1996), an ideological narrative that in the case of terrorist and violent organizations legitimizes violence. Such a narrative could be extraordinarily appealing after suffering a loss of personal significance or meaning, when people usually experience a thirst for revenge against those they consider blameworthy (Kruglanski and Orehek, 2011). By virtue of being part of a violent group and the adoption of its narrative, the use of violence that is generally reprimanded becomes tolerable (Bélanger et al., 2019).

Another motive that could explain why some individuals join these violent groups is identity fusion, or the development of a feeling of visceral sense of connection with the group (Swann et al., 2012). One of the key characteristics of violent and terrorist groups is that their members are willing to fight and even die for the group, and identity fusion research has consistently confirmed that fusion is a successful predictor of such extreme actions (see Gómez et al., 2020 for a review). Up until now, two main mechanisms have been identified as a cause or an amplifier of fusion with a group: shared experiences with other individuals, particularly dysphoric experiences (e.g., Whitehouse et al., 2017), and shared values (e.g., Swann et al., 2014). Of particular interest here is the fact that individuals might even fuse with groups that they do not (yet) belong to and with whom they do not share any previous association, such as when they perceive that the negative treatment suffered by an outgroup clashes with one’s own beliefs (Kunst et al., 2018). Examples of fusion with a group have been found among Libyan insurgents fighting against the Gaddafi regime (Whitehouse et al., 2014), captured ISIS fighters (Gómez et al., 2017), Pakistani participants supporting the Kashmiri cause (Pretus et al., 2019), supporters of an Al Qaeda associated group (Hamid et al., 2019), Northern Irish loyalist and republican paramilitaries (Ferguson and McAuley, 2020), and fighters against the Islamic State including Peshmerga, Iraqi army Kurds, and Arab Sunni Militia (Gómez et al., 2017).

Although there is an impressive number of theoretical models on the causes of violent extremism (e.g., Neumann and Kleinmann, 2013; Hafez and Mullins, 2015; Pisoui and Ahmed, 2016; Vergani et al., 2018), less common are investigations including empirical data about this issue. A recent qualitative examination of the themes explaining why people join terrorist groups (i.e., ISIS and Al-Qaeda) in Kuwait through interviews with prison inmates identified five reasons for involvement: religious identity development (progression of the religious identity), personal connections (development of close social bonds with individuals and religious organizations), propaganda (influence by social media), defense of Islam (perception that Islam and specifically the Sunni sect of Islam is under threat), and social marginalization (social risk factors) (Scull et al., 2020). Although this model is promising, one of its limitations is that it is based on the analyses of interviews with members of terrorist groups that are focused on ideological factors. Terrorists from groups with a different focus than ideology or from groups with a similar focus but in different contexts might decide to embrace such groups for reasons not captured with this sample. For instance, some authors have suggested that the reasons for entering into terrorist groups differ in conflict zones (i.e., trauma and revenge) and non-conflict zones (i.e., discrimination, marginalization, frustrated aspirations, desire for adventure, romance, personal significance, or the desire to be heroic) (Speckhard, 2015). Another limitation of this model is that it is based on interviews with only nine terrorists, so its generalizability is questionable.

While the previous models have contributed enormously to the identification and systematization of the reasons leading to involvement in violent groups, they have stopped short of providing an overarching scheme that explains how the various factors relate to one another. Another important limitation is that most of these classifications have not been supported by empirical data (see Scull et al., 2020 for an exception). In other words, previous research has not tested whether the classification is valid for groups with diverse organizational structures and whether the reasons for joining specific types of terrorist groups differ.

Our goal here is to take a preliminary step toward developing an overarching scheme informed by empirical data. At a very general level, the approach we suggest is reminiscent of the time-honored distinction within social and personality psychology between explanations of nature vs. nurture, genetics vs. environment, or traits vs. situations (e.g., Mischel, 1968). In a more specific sense, our approach draws on a classic theme in the social influence literature first advanced by Kelman (1958). He distinguished two forms of attitude change, one produced by internalization and the other produced by compliance. In the present context, we argue that internalization occurs when people are drawn into terrorist groups by the fit between the group and personal qualities such as identities, ideologies, narratives, needs, grievances, or background characteristics. It comprises an ample variety of motives that include, among others, the pursuit of power, status, and the desire to become a hero (e.g., Kruglanski et al., 2019); the establishment of close relational bonds with others (e.g., Gómez et al., 2019); and the adoption of highly valued causes (e.g., Atran, 2010). In contrast, compliance occurs when people are compelled to enter the group due to features of the situation, most notably propaganda, threats, or other situational pressures.

Although some authors have discussed compliance and internalization as potential reasons for joining violent groups (e.g., McCauley and Moskalenko, 2008), no research to date has systematically studied the role of these processes in the decision to join such groups. To determine the viability of this approach, we sought to identify terrorist groups in which either compliance or internalization seemed likely to emerge.

For evidence of the role of compliance, we were guided by a report by the United Nations Office on Drugs and Crime (UNODC, 2018), which indicated that forced recruitment is especially high in Africa and Asia (see Becker, 2010). For example, the Liberation Tigers of Tamil Eelam (LTTE) has been accused of forced recruitment of children, especially after 2002 (Ramesh, 2004).

For evidence of internalization, we referred to accounts of religious terrorist groups such as ISIS who are renowned for recruiting followers in mosques, prisons, and through social media sites in Western democratic countries (Berger, 2015).

Given these accounts, we selected a sample of former LTTE members and a second sample of former Islamist terrorists (mainly ISIS and Al-Qaeda members) for the current research. We expected to discover more evidence of compliance among former LTTE members than former members of Islamist groups. Conversely, we also expected to find more evidence of internalization among former members of Islamist groups than former members of LTTE.

## Overview of the Current Research

To test our predictions, we examined two groups that varied in ideology, nationality, and type of radicalization. Study 1 analyzed ex-members of the Liberation Tigers of Tamil Eelam (LTTE), a ruthless ethno-nationalist separatist terrorist group, proscribed by 32 countries as a terrorist organization (including the European Union, Canada, the United States, and India). The LTTE is the only terrorist group that has assassinated two serving heads of state using suicide bombers (the Prime Minister of India in 1991 and the President of Sri Lanka in 1993). All the participants interviewed in Study 1 were Asian.

Study 2 focused on Islamist radicals who, at some point, were members of violent jihadist groups. These groups included ISIS, Al-Qaeda, or one of their associated organizations that can be considered part of the global jihadi movement. All groups associated with the global jihadi movement oppose liberal democracies and are in favor of authoritarian religious oligarchies ruled by a fundamentalist interpretation of Sharia (Islamic law). While some of these groups believe in nationalism in the short-term, all of them ultimately seek to establish a borderless worldwide Caliphate in the long-term. In addition, these groups consider violent offensive jihad (Holy War) as the only way to achieve these goals. They also claim that it is incumbent upon all Muslims to engage in or facilitate this holy war. Most of the participants interviewed in Study 2 were European.

We pooled analyses for the protocols from either semi-structured interviews (Study 1), or from narratives derived from audio recordings (Study 2). Based on our research questions, the characteristics of the studies, and the nature of the data obtained, we combined data-driven coding in the First Cycle (descriptive coding method) with theory-driven coding in the Second Cycle (theoretical coding) that allowed us to refine our initial categorization (for a discussion of coding methods see Saldaña, 2013). After an initial review of the data using a descriptive coding method, we extracted specific codes for each participant. Such codes were labels –words that reflected the main topic of the reasons to embrace the radical group– such as force, propaganda, family issues, personal issues, and/or ideals. This first descriptive coding revealed two main patterns: internal forces (i.e., reasons related to the individual that push to the radical group, that based on Kelman, 1952, correspond to identity-related reasons or internalization) and external forces (i.e., reasons related to external sources that pull the participant toward the radical group, that based on Kelman, 1952, correspond to influence or conformity reasons or compliance). These two categories were subdivided into subcategories. We elaborated three identity-related reasons for joining terrorist groups that reflect different forms of internalization (influences on personal, relational, and collective identities), and a second cluster of three reasons that involved compliance (charismatic persuasion agent, propaganda, and coercion). *Personal identity* refers to those aspects of the self-concept that allow differentiation from all others and make us unique; r*elational identity* is derived from connections with significant others and encompasses one’s roles in close relationships; and *collective identity* comprises the cognitions, emotions, and values strongly linked to group membership. Compliance through a *charismatic persuasion agent* refers to being convinced by an individual group member such as a radicalized Imam cleric or a professional recruiter; *propaganda* refers to being convinced to join by recruitment material such as videos on the Internet; and *coercion* refers to being taken into the group by force. With the foregoing theoretical framework in mind, two judges recategorized the reasons in a second cycle coding, and then, intercoder agreement was evaluated. Then, frequency counts were presented for each category and subsequent subcategories and they were ordered in a hierarchical way with typical exemplars. Chi-squared tests were used to compare pairs of percentages within groups, and *z*-score tests were used to compare proportions between groups. What follows is a presentation of the methodology and results of each individual study.

### Study 1. Why Ex-Members of the Liberation Tigers of Tamil Eelam (LTTE) Joined the Group

The LTTE’s guerilla and terrorist activities were targeted at achieving a mono-ethnic separate state for the Tamil people in Sri Lanka. Upon its foundation on 5 May 1976, the LTTE commenced its campaign for a separate state. The murder of the Tamil Mayor of Jaffna, Alfred Duraiappa, in 1975, was the LTTE’s first assassination and was conducted personally by Velupillai Prabhakaran, the leader of the LTTE. The LTTE was a well-developed terrorist group that operated an overt/semi-covert political wing and a clandestine military wing. Over time, the LTTE developed capabilities in guerrilla and mobile warfare but continued to employ terrorism until the end of the movement. They even developed affiliations with outside organizations, both within and beyond the theater of conflict, to establish a support base and ensure a steady stream of funding. The LTTE was finally defeated militarily in May 2009. The Sri Lankan government launched a formidable rehabilitation program to reintegrate the majority of the former members of the LTTE into the community. However, while the LTTE’s operational capability on the ground has been neutralized, LTTE’s overseas networks remain intact, and continue to pose a threat to Sri Lanka. Study 1 aimed to understand the reasons that a sample of ex-Tamil Tigers gave for joining this terrorist group.

#### Method

##### Participants

Seventy-five ex-members (38 women and 33 men; four did not report sex) of the LTTE were interviewed by a member of the research team. Their age varied from 22 to 56 with a mean age of 34 (*SD* = 7.82). Seventy-three had Sri Lankan nationality (two did not report nationality). Most of them were of Tamil ethnicity and Hindu. Only forty-four of them gave reasons for joining the group and were included in the analyses.

##### Procedure

Interviews were conducted in Kilinochchi and Viswamadu community centers, two regions where former LTTE members were reintegrated. The sample was selected randomly from a group of former LTTE members during community follow-up visits by the researcher. Community leaders gathered all the former terrorists who were available to participate in the study during the community visits. The data were collected using a structured questionnaire. Respondents were asked “How did you or others come/happen to join the LTTE? (What were the key reason that encouraged others/you to join this group?).” Because we were interested in the main reason for joining the group, participants were asked to think and choose only one, so the reasons showed in the result section are mutually exclusive. To diminish social desirability bias, the interviewer used third-person language instead of second-person language when discussing highly sensitive topics.

After the interview, two judges read all of the reasons provided by the participants and decided which pathway aligned with each given reason. They could discuss preliminary disagreements as needed. The reasons that didn’t fit in with any of the pathways were classified as *other*.

#### Results

**FIGURE 1:**
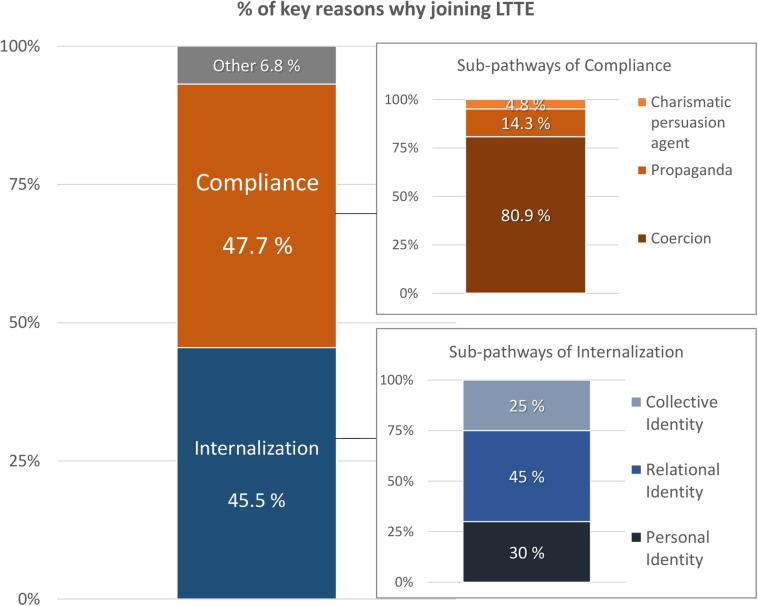
Percent breakdown of key reasons that former members joined LTTE.

Judges showed a complete agreement in the sub-pathways of collective identity, relational identity, propaganda, and charismatic persuasion agent (*k* = 1), and an adequate inter-judge agreement in the sub-pathways of personal identity (*k* = 0.83), and coercion (*k* = 0.86). Those three reasons where there was disagreement were categorized as other^1^. Figure 1 shows the percentage of the key reasons why participants joined LTTE. Ex-fighters from LTTE expressed an equivalent number of reasons for compliance versus internalization, χ^2^(1) = 0.02, *p* = 0.876. Within sub-pathways of compliance, more participants expressed reasons related to coercion than propaganda, χ^2^(1) = 9.80, *p* = 0.002, or charismatic persuasion, χ^2^(1) = 14.22, *p* < 0.001. Within sub-pathways of internalization, there were no differences in the percentages of participants who expressed reasons related to personal, relational or collective identity.

Almost half of the participants mentioned some form of compliance as the key reason for joining (47.7%). Looking at the compliance pathway, most identified *Coercion* as the main reason for entering the group (80.9% of the total reasons referred to compliance). Some examples of coercion are (“P” refers to the participant number): P14 remembered joining “By force when going to school”; P21 told that she “did not like to join but had to” because “one in every family joined”; P50 explained he joined “When LTTE forcefully gathered people”; and P60 told she joined because of death threats by LTTE.

*Propaganda* was mentioned by a 14.3% of participants. Some examples of propaganda are: P25 mentioned different kinds of publicity by LTTE; P38 mentioned “Street drama of LTTE media”; and P44 referred to “Publicity, street drama, video” and “LTTE publicity.”

*Charismatic Persuasion Agent* was mentioned by a 4.8% of participants. An example was P3, who talked about politicians highly valued by the community who recruited them.

Approximately half of the participants gave reasons for joining related to internalization (45.5%). Around half of these participants referred to *Relational Identity* as the reason for joining the group (45%). The examples for this sub-pathway refer to the loss of relational ties as a reason for becoming part of LTTE: P1 recognized having joined because people he knew died; P10 referred to losses and displacement; P15 remembered joining when his family died; P40 joined after his mother died; P49 joined because of loss of relatives; and P56 declared he joined after his wife’s death.

*Personal Identity* was mentioned by 30% of participants who referred to internalization. Some examples are: P4 mentioned “Not much education,” whereas P5 talked about the “Bad situation around us” as reasons for joining. P8 recognized having a very hard life and P59 joined because she was systematically neglected from jobs.

Finally, *Collective Identity* was mentioned by 25% of those who referred to internalization. Examples are: P12 said “The attachment I have about my ethnicity”; P13 “Thought we wanted a Tamil nation”; P19 joined “To get rights for Tamils”; P73 did it “to fight against discrimination and differences in social status, class.”

#### Discussion

Study 1 shows that when we asked former LTTE about their main reason for joining the group, around half of them mentioned compliance while the other half referred to internalization. In the case of compliance, most participants explained that they joined the group because of coercion, some of them because of propaganda, and almost none because of the influence of a charismatic leader. However, in the case of internalization, the motives referring to the different sub-pathways were more balanced. The loss of relational ties such as, for example, the death of family members, was a key reason that encouraged joining LTTE. However, personal and collective identity were also mentioned as reasons for joining the group.

One of the limitations of this study is that former LTTE members were instructed to report “the key” reason that encouraged them to join. This procedure does not allow for the possibility that several, instead of just one, factors motivated them to enter the group. That is, complex social phenomena, such as entering violent groups, are often due to multiple causes acting together (e.g., Vergani et al., 2018; Atran, 2020). To learn more about the full range of considerations that led people to join violent groups, in Study 2, we recorded life stories of members of radical Islamist organizations to identify all the myriad reasons that drove them to embrace violent groups as opposed to just the most important reason.

### Study 2. Why Islamist Radicals Joined the Group

Study 2 analyzed the life stories of twenty-one Islamist radicals who were, at some point in their lives, members of violent jihadist groups. These groups included ISIS, Al-Qaeda, or one of their associated organizations that are considered part of the global jihadi movement.

#### Method

##### Participants

A total of 21 participants (18 men and 3 women, ranging in age from 21 to 59 years) qualified for this study by indicating that they had been a member of a jihadist terrorist organization at some point in their lives. There were no age, gender, or nationality criteria pre-established. Most participants were European. Six participants were Belgian, another three were Belgian-Moroccan, four participants were from Britain and three from France. Single individuals were Belgian-Tunisian, Pakistani-Spanish, Kosovan, Egyptian, and German.

##### Procedure

A member of the research team interviewed participants and then created life stories based on each interview. The way each participant was recruited for the interview varied person-to-person. In some cases, the participant was introduced to the researcher by a social worker or a community member. Sometimes, it was another participant who introduced the researcher to the next participant following a snowball technique. Other times, a friend or a family member introduced the participant. On some occasions, a lawyer introduced the participant, or the researcher contacted the participant online and arranged a face-to-face meeting.

The locations of the meetings were as diverse as the recruitment method. Some interviews took place in a lawyer’s office with the participant’s attorney present. Other times, they took place in the participant’s domicile with no one else present. Lastly, some of the interviews were conducted in cafes or parks. All participants were told that the purpose of the interview was to attain their life history to show how and why they joined the Islamist group. They were informed that this research would be used for academic publications and that their identities would be anonymized. After oral consent was obtained, the researcher followed a semi-structured questionnaire. In some cases, there were multiple meetings with the same participant. The interviews took two hours on average and all responses were handwritten by the researcher.

The researcher gathered all the information of the life stories of each participant and then recorded a clip-summary of each life story separately. Then two members of the research team listened to the recordings and did a first round of coding by discussing the pathways that aligned with the reasons given for joining the Islamist groups. We organized the reasons for joining these violent Islamist groups into the same pathways as in Study 1: compliance (charismatic persuasion agent, propaganda, or coercion), and internalization (influences on personal, relational, or collective identities). Then two independent judges categorized the reasons given within the life stories of why participants joined the terrorist groups. They were offered the possibility to discuss preliminary disagreements. It was decided whether the reasons of each participant *did* or *did not* pertain to each of the pathways presented by indicating *yes* (coded 1) or *not* (coded 0) in each rationale. Reasons where disagreement was found were then rated as *other*. It is important to note two key differences in methodology between this and the previous study. First, in Study 1 we asked participants directly about the reasons for why they had joined the group, whereas in Study 2 this information emerged spontaneously during the conversation. Second, participants could only give one reason for why they joined the group in Study 1; in Study 2 they were able to give multiple reasons.

#### Results

The inter-judge agreement was complete for the sub-pathways of personal identity, relational identity, charismatic persuasion agent, and propaganda (*k* = 1.00). The agreement for collective identity was acceptably high (*k* = 0.89). There were no reports of coercion in this sample. Each life story included several reasons that could explain why participants joined radical groups. This study did not include one unique reason, but several, as the process of radicalization is complex and might entail different sources of influence throughout the life of an individual. So, contrary to what was reported in Study 1, where the total number of reasons was equivalent to the total number of participants, in Study 2 the 21 participants gave a total of 60 different reasons for joining the terrorist group. Many life histories contained elements with overlapping themes. For example, 16 life stories included reasons related to personal identity, but some of the same life stories also included reasons related to relational identity, collective identity, or some kind of social influence. The internalization pathway included a total of 47 reasons, with 16 life stories including personal-identity reasons, 17 included relational-identity reasons, and 14 included collective-identity reasons. A total of 13 reasons were considered evidence of compliance, with 8 life stories including reasons related to the presence of a charismatic agent and 5 including some form of propaganda. To transform the percentage of life stories where a reason was present (e.g., internalization) to the percentage of that specific reason among the total number of reasons presented in the life stories, we considered the total number of reasons offered as 100% (*n* reasons = 60) instead of the total number of participants/life stories analyzed (*n* = 21). So, the 47 reasons related to internalization corresponded to 78.3% of the total reasons present in the life stories. As in Study 1, the percentage of the subcategories took the total number of reasons in each category to be 100%. Please see Table 1, for reconversion values for both studies.

**TABLE 1:** Percentage of reasons among total reasons.

LTTE ex-fighters			Ex-Islamist radicals		
*Reasons for joining*	*n*	*%*	*Reasons for joining*	*n*	*%*
Total	44	100%	Total	60	100%
Compliance	21	47.73%	Compliance	13	21.67%
Internalization	20	45.45%	Internalization	47	78.33%
***Compliance***	***n***	***%***	***Compliance***	***n***	***%***
Total	21	100%	Total	13	100%
Charismatic agent	1	4.76%	Charismatic agent	8	61.54%
Propaganda	3	14.29%	Propaganda	5	38.46%
Coercion	17	80.95%	Coercion	0	0%
***Internalization***	***n***	***%***	***Internalization***	***n***	***%***
Total	20	100	Total	47	100
Personal Identity	6	30%	Personal Identity	16	34.04%
Relational Identity	9	45%	Relational Identity	17	36.17%
Collective Identity	5	25%	Collective Identity	14	29.79%

**FIGURE 2:**
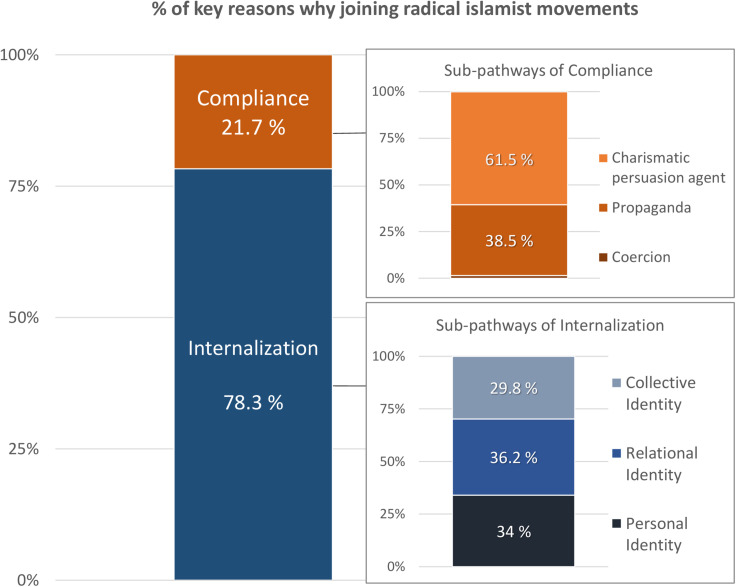
Percent breakdown of key reasons that former members joined Islamist terrorist groups.

Figure 2 shows the percentage of life stories in Study 2 where the specific reason (compliance versus internalization) was mentioned. For each of these two pathways, the percentage of reasons that referred to each of the corresponding sub-pathways were listed. Note that for clarity, we are reporting the results here in the same format that we did in Figure 1. However, the data collection process was different in that participants in Study 1 reported only the single most important reason for joining, whereas participants in Study 2 reported all the reasons that came to mind. Overall, life stories in Study 2 included more reasons related to sub-pathways of internalization (a total of 47 reasons) than reasons related to compliance (a total of 13 reasons), χ^2^(1) = 19.27, *p* < 0.001.

Over 80% of the life stories analyzed included some kind of internalization as the key reason for joining the group. Regarding the sub-pathways of internalization, about one third of the reasons reported by participants referred to *Relational Identity*, such as disappointments with the close family that deteriorated their relational ties: P1 was very upset with her father, her family was disappointed at her, and she ran from home; P4 experienced feelings of exclusion and isolation from his family and his community. P4’s family and community did not understand him from the beginning, and he remained isolated; P8 showed an unhealthy family relationship, and he was looking for a home, a place to belong; P10 also came from a broken home (i.e., his parents got divorced when he was very young, he had an absent father who was unable to help him), he had a big network of Moroccan friends, with whom he felt oneness and who satisfied his need to belong. One of his friends died, and, during the funeral in the mosque, he had a transformative experience and realized that he wanted to be part of the religious community. The group of Jean Louis Denis (a recruiter who convinced others to go to Syria to fight against the Syrian government) became a kind of family to him. The idea of going to Syria was important to him because he thought that there, he would be offered a family, a wife, a home, and the support necessary to sustain them; P11 also came from a broken home (i.e., divorced parents) and experienced tension with his parents, including lots of conflicts with his father. He went to Morocco to see some friends and he felt a sense of belonging. Finally, he went to Syria with his friend; P16 also came from a broken home and had experienced losses and divorce. She got in touch with a man from Syria online and initiated a virtual romance with him. She later converted to radical Islam to be with him and to marry him. Another participant, P9, mentioned that during a stay in prison he found a group of radical Islamists who were willing to accept him; he established close relational ties with members of a terrorist group which allowed him to overcome his feelings of social isolation.

Approximately another third of the reasons reported by participants refer to examples of setbacks or advancements of their personal identity. P1, for example, used to live in the street after leaving her home, she had a “wild life,” no self-respect and feelings of desperation. She wanted personal recognition and looked for redemption. P2 had depression and emotional problems and found in radical Islam an escape from depression; she also wanted to be part of something exciting. P6 saw in Syria an opportunity to become someone important: to be a hero. P11 was very overweight and had been teased because of that. P16 was looking for a change in her life. P15 had problems with the law. P19 has been kicked out from school and has an aggressive personality.

Finally, 29.8% of the internalization reasons included references to collective identity in terms of Muslim identity or sharing values and important ideas with a radicalized group. For example, P3 wanted to live a conservative religious life. P6 wanted to help Syrians because he believed that his own group (Kosovans) had lived through something similar in the 1990s. P7 and P15 mentioned problems with the “new world order.” P11 was committed to ideas such as liberating Palestine and feeding refugees. He really wanted to embrace the Islamic identity, and he was very politicized. P12 was committed to the idea of defending and standing up with other people to fight against the discrimination of Muslims. Born from a white Belgian mother and a Moroccan father, he had some identity conflict issues. He was an Arab in Belgium and a White in Morocco. He was looking for a new, broader, and clearer collective identity. Feeling oppression and racism in both countries, he was really attracted to the idea of a Muslim Ummah.

On the other hand, less than a quarter of participants reported reasons related to compliance as a pathway for joining the group (21.7%). When looking at the sub-pathways of compliance, about two thirds of their expressions (61.5%) referred to the influence of a charismatic persuasion agent. For example, P1 was deeply influenced by an Arabic teacher who helped refugees. P3 was persuaded by neighbors, and, presumably by P2 (who was his wife). P10, P11, and P13 were politicized by Jean Louis Denis, the charismatic leader mentioned before, who encouraged them to go to Syria to show that they were real Muslims by trying to stop the humanitarian crisis of the refugees by combating its true causes. P20 met this top recruiter in Brussels as well and he became radicalized.

The other third of reasons related to compliance referred to propaganda (38.5%) that in most cases was combined with the influence of charismatic leaders. For example, P2 was recruited by her neighbors as well as by watching videos on internet. P4 met an Imam who influenced him, in addition to watching propaganda videos. P12 met an old colleague, the son of a radicalized Imam, who put ideas in his mind about what it meant to be a true Muslim. Afterward he and his friends began to watch videos of propaganda. No examples of coercion were identified in the life stories of the former Islamist terrorists.

#### Discussion

When we analyzed the main reasons that former Islamist terrorists spontaneously gave for joining their terrorist group, results indicated that most examples referred to the internalization pathway. Here, the distribution of the reasons in the three sub-pathways was quite evenly balanced between examples referring to relational, personal, and collective identity. Less common were examples of the compliance pathway, which usually corresponded to the influence of a charismatic leader combined with propaganda.

### Additional Analyses

Although the procedure of Study 1 and Study 2 was different, we sought to make rough comparisons between them by transforming the original percentage of participants in Study 2 to make it comparable to Study 1 (see Table 1). We then compared the proportions of specific reasons for each group using a *z*-score test. Ex-Islamist radicals showed significantly more internalization reasons (47 over a total of 60 reasons) than LTTE ex-fighters (21 over a total of 44 reasons), *z* = 3.46, *p* < 0.001. The opposite pattern was found for compliance, with LTTE ex-fighters offering more reasons regarding compliance than ex-Islamist radicals, *z* = 2.80, *p* = 0.005. More specifically, within the compliance reasons, LTTE ex-fighters showed more reasons related to coercion than Islamists, *z* = 5.26, *p* < 0.001, whereas Islamists offered slightly more reasons related to a charismatic persuasive leader than LTTE ex-fighters, *z* = 1.98, *p* = 0.048. However, there were no differences between groups in the proportion of propaganda, *z* = 0.29, *p* = 0.772. Within the internalization category, there were no differences between LTTE ex-fighters and Islamists in the proportion of reasons related to personal, relational, or collective identity.

## General Discussion

The current research provides empirical evidence regarding why people enter terrorist groups. Specifically, in two studies former members of terrorist groups were asked for either their primary reason for joining (Study 1, former LTTE members), or for their life narratives in which they spontaneously referred to reasons for joining (Study 2, former members of radical Islamist groups). Mindful of the classic distinction in attitude-change literature advanced by Kelman (1958), we inspected participants’ responses. We identified two pathways through which people may join violent groups: compliance and internalization. Compliance occurred when individuals joined groups because they were persuaded by a charismatic persuasive agent, exposed to propaganda, or coerced. In contrast, internalization occurred when individuals joined groups because of a convergence between the self and the group associated with their personal, relational, or collective identities.

The results of these two studies offered empirical evidence in line with our hypotheses. As expected, compliance was more frequently cited among former LTTE members than among former Islamist radicals. While almost half of former LTTE members reported compliance as a reason for joining the group, Islamist radicals cited compliance much less frequently. Also consistent with our expectations, former members of Islamist groups cited internalization more frequently than former LTTE participants: while more than three quarters of the reasons given by Islamist radicals for why they joined the group referred to internalization, less than half of former LTTE participants reported that this was a motive for joining.

As we have seen, a sizeable proportion of LTTE members were forced to join through coercion. As a consequence, we notice that some of them, even if they had been engaged in the radical group, were not cognitively radicalized. This was the opposite of our sample of Islamist radicals, who embraced the importance of the “cause” (collective identity). These findings confirm Borum’s (2011) contention that the process of radicalization is not necessarily the same as the process of action pathways, and that some members of terrorist groups can commit violent actions without being deeply ideologically radicalized. Whereas LTTE members were forced to enter in the group by coercion, Islamist radicals were persuaded by propaganda, which can explain why Islamist radicals show more cognitive radicalization than LTTE members. Another relevant finding is that personal identity reasons were more important for Islamist extremists than for LTTE members. This finding was not surprising given that most members of LTTE were forced to join, which could explain the relative powerlessness of their group to fit their personal identity. This confirms what has been commonly highlighted in the context of violent extremist research: non-identical root causes might apply to different types of terrorism and to the same types of terrorism in different contexts (e.g., Rapoport, 2004; Noricks, 2009; Speckhard, 2015). It is necessary to note as well that most of the former Islamist extremists that we interviewed were European, whereas most LTTE members were Asian, which is consistent with Vergani et al. (2018) conclusions that personal factors play a more prominent role in Europe, North America, and Australia than in the rest of the world.

Previous research might support why internalization in general, and personal identity in particular, is a relevant factor for joining Islamist radical groups. Although persuasion and propaganda are also important for understanding Islamist radicalization (e.g., Gendron, 2017; Kruglova, 2020), people do not become Islamist radicals through mere coercion or brainwashing (Sageman, 2004, 2008). Islamist terrorists typically go through a process involving active and selective engagement with groups that fit their idiosyncratic characteristics, thus suggesting internalization (Chernov-Hwang and Schulze, 2018; Scull et al., 2020). Other examples of internalization might be the research by Scull et al. (2020), indicating that participants in their study experienced a process in which religion became a central part of their personal identity. As their religious identity developed, they met people involved with Al-Qaeda or ISIS who, in turn, exposed them to propaganda in support for the radical ideology (see also Dawson and Amarasingam, 2017 who suggest existential concerns and religiosity). And some other investigations suggest that establishing relational bonds and relationships with members of Islamist terrorist groups are the common thread encouraging entry as well as in fostering commitment (Chernov-Hwang and Schulze, 2018).

Taken together, the present studies make a series of theoretical and empirical contributions to previous research regarding the reasons for entering into terrorist groups. First, we have introduced a new way of conceptualizing the reasons why people enter violent groups that draws on classic work on attitude change (Kelman, 1958). Our conceptualization is also based on an extensive review of the main theoretical models on the causes of engagement in terrorist groups, including the 3N model (Kruglanski et al., 2018), the model of the three Ps of radicalization (Vergani et al., 2018), and the model developed by Hafez and Mullins (2015), among others. By integrating the insights offered by these approaches, our conceptualization offers a new lens through which to contemplate the reasons that motivate individuals to join violent groups. Our conceptualization also makes it possible to establish distinctions between different types of terrorist groups that have been not considered until now. We believe that these distinctions will be useful for explaining why and how people decide to enter terrorist groups and for identifying the people and circumstances which are at high-risk for the creation of more adherents to a terrorist group.

Second, most of previous research on the causes that explain why individuals join terrorist groups is based on theoretical approaches to the phenomenon and does not satisfy the minimal methodological and empirical requisites of rigorous science (Neumann and Kleinmann, 2013). At an empirical level, for instance, studies have usually relied on secondary sources, opportunistic interviews, and even anecdotal evidence to support their arguments; investigations including samples of current and former terrorists have been inappropriately scarce (e.g., Neumann and Kleinmann, 2013). As a result, there is a huge quantity of concepts and theoretical models that are not backed up by tangible evidence within the field, which has prompted some experts to make a call for more scientifically-grounded research on why people join terrorist groups (e.g., Schuurman, 2018). Our studies responded to this call by including two samples of former terrorists and, as such, they increase our confidence in the possibility that the different pathways and sub-pathways leading to engagement with violent extremist groups that we have established with our model are a true reflection of this process.

Third, our research also may be useful for designing cost-effective strategies to counter violent extremism and, more specifically, to prevent people who are not yet members of terrorist groups from joining them. Our results indicate that factors related to compliance and internalization play a determining role in this process and that their relative importance vary as a function of the type of terrorist group along with the context in which the groups operate. This could help us design preventive interventions tailored to the specific characteristics of different terrorist groups and socio-political circumstances in which these interventions are meant to be applied.

When dealing with groups or contexts in which internalization predominates as a reason for joining, these strategies should be aimed at fighting feelings of discrimination, marginalization, and social alienation so that people from populations that are at risk may experience a better fit between themselves and groups that do not support violence. This goal can be achieved in several ways, such as advancing community-aimed educational interventions (RAN, 2019), promoting the values of tolerance, solidarity and acceptance (RAN, 2019), or running interventions aimed at the development of feelings of brotherhood toward non-violent people through the practice of sport, like the London Tiger group has been doing in the United Kingdom for more than a decade (National Academies of Sciences Engineering and Medicine, 2017). People are often looking for new groups that allow them to satisfy their personal needs, to engage in meaningful relational roles, and to feel that there is a noble and legitimate cause behind their actions. When non-violent groups are able to provide these things, people may be more open to joining the ranks of such groups even though they do not commit violent offenses (e.g., Atran, 2010, 2020).

On the other hand, when we approach groups or circumstances in which compliance is more important than internalization as a reason for joining, the specific strategies that we should use will depend on the sub-pathways through which compliance exerts its effects. If people join terrorist groups mostly through propaganda and charismatic influence agents, strategies aimed at increasing resistance to persuasion, like the diffusion of counter-narratives, educational interventions to increase individuals’ critical thinking, or public discrediting of terrorist leaders by former terrorists should be particularly effective (RAN, 2019). However, although some research focused on ISIS supports the positive effect of counter-narratives, there is also evidence that counter-narratives can have counterproductive effects on sympathizers of ISIS and individuals at great risk of radicalization, and regardless of the source of the message all counter-narratives with a religious argument backfired (Bélanger et al., 2020). If people join because of coercion, “hard” measures, like the decapitation of terrorist organizations, that is, the killing or imprisonment of terrorist leaders, may be needed (Price, 2012).

Lastly, our studies highlight some potential future lines of research. First, future investigations could test whether our conceptualization applies not only to ethno-nationalist separatist and religious terrorists but also to single-issue, left-wing, and right-wing violent extremists by examining the relative importance that compliance and internalization have in these different groups. Given the upsurge of terrorism from the radical right that has occurred in the last decade in some Western countries (Atran, 2020), we think that a deep exploration of the reasons that are driving people to join right-wing extremist groups at increasing rates would be particularly advisable. Second, other studies could test our model with violent groups that do not fall under the umbrella of terrorism, like Latin gangs or criminal organizations like the mafia, and compare them to terrorist groups. As gang members are more motivated by friendship, affiliation, and personal interest and less motivated by ideological causes than terrorists (Decker and Pyrooz, 2011), we think that internalization via personal and relational identity fit may be more frequent among gang members than among terrorists and, conversely that internalization via collective identity may be more common among terrorists than gang members. Third, some longitudinal studies could be run to gain a better understanding of how the process of joining violent extremist groups unfolds in real-time and to discover the different ways in which the factors covered by our model interact and influence the end result of this process. It is possible, for instance, that charismatic influence agents and propaganda mutually reinforce the impact of the internalization sub-pathways, thus making individuals more prone to becoming terrorists.

## Limitations

The present research has some limitations. In particular, the different results obtained in the two studies could be due to methodological differences as opposed to the intrinsic characteristics of the groups (i.e., LTTE members were asked about the main reason for joining the group, whereas Islamist radicals recounted their life stories and the reasons for joining were extracted from the narratives).

Another potential limitation is that former terrorists may be concerned with presenting themselves in a favorable light that is not particularly accurate, which raises concerns about the validity of their reports. In particular, the interviewees may adjust their responses to give a good impression of themselves or the group, to appear less responsible for their actions and decisions, or to preserve their positive self-image. After all, former terrorists tend to overemphasize the role of situational/external factors such as persuasion, coercion and duty in explaining their involvement to dilute their own culpability (Horgan, 2014). They are also inclined to downplay the role of personal motives such as need for power, status, and thrill-seeking, which are rarely expressed in interviews (Horgan, 2014). These issues are especially notable in Study 1, where participants were explicitly asked for their reasons for joining the group. Although some researchers have found that terrorists are sincere in their answers (Kruglanski et al., 2019) and others have argued that it is necessary to take terrorists accounts of their motivations seriously (Nilsson, 2018; Dawson, 2019), we need to be cautious when interpreting interview data from terrorists or we run the risk of over- or under-stating the significance of certain experiences and events (Horgan, 2012, 2014).

Also, terrorists go through a dynamic and transformative process as they move along the different stages of radicalization and engagement. Their explanations of their reasons for joining the group may differ depending on their stage of (dis)engagement (Horgan, 2012). There is no reason to suppose that the explanations offered at one particular stage should be taken as more valid than those given at others (Dawson, 2019). Furthermore, as our main research interest is extreme behavior, our focus has been members of two of the most violent groups in history, whose members are willing to kill (and maybe some of them actually did it) and die if necessary, for the group or for their convictions, whether the categories that we have used here would apply to non-violent groups is an empirical question that opens the door for future research. Finally, the samples were quite small. Future research should assess the generalizability of our findings.

To address these limitations, future researchers might consider: (1) using the same methodology for data collection independently of the group and the stage of radicalization; (2) making use of sophisticated coding and analysis techniques (Miles et al., 2019); (3) combining qualitative and quantitative research methods (White, 2000); (4) collecting data with people at different stages of radicalization; and (5) comparing and verifying the data obtained from interviews with other data sources, such as the penitentiary and judicial records.

## Conclusion

As the UN has acknowledged (United Nations, 2006), measures and policies aimed at countering violent extremism should focus on the prevention of radicalization among members of vulnerable communities. To this end, we need to understand the reasons that drive individuals to join violent extremist groups (e.g., Bakker, 2015; Schuurman, 2018). With the present research, we have attempted to integrate classic socio-psychological research on attitude change (Kelman, 1958) with more contemporary approaches to the study of terrorism (e.g., Kruglanski et al., 2018; Vergani et al., 2018). We report two studies with former members of terrorist groups that offer empirical support for our conceptualization that reasons for joining terrorist groups fall under the categories of internalization or compliance, which in turn can further be broken down into easily identifiable sub-pathways. It is our hope that this new theoretical frame will provide new insights into how to prevent violent radicalization as well as foster de-radicalization.

## Data Availability Statement

The raw data supporting the conclusion of this article will be made available by the authors, without undue reservation.

## Ethics Statement

Ethical review and approval was not required for the study on human participants in accordance with the local legislation and institutional requirements. Written informed consent for participation was not required for this study in accordance with the national legislation and the institutional requirements.

## Author Contributions

ÁG designed the study. ÁG, WS, AV, JC, and FM wrote the manuscript. MH and NH collected the data. MM, LL-R, and BP analyzed the data. All authors contributed to the article and approved the submitted version.

## Conflict of Interest

The authors declare that the research was conducted in the absence of any commercial or financial relationships that could be construed as a potential conflict of interest.

## Footnotes

1 Three reasons (P27: LTTE being there –everywhere; P61: LTTE was always there; and P62: When I was arrested by security) were categorized as “other” due to disagreements in the categorization process.
